# Longitudinal Development of Primary and Secondary Posttraumatic Growth in Aging Veterans and Their Wives: Domain‐Specific Trajectories

**DOI:** 10.1002/jts.22331

**Published:** 2018-10-19

**Authors:** Rahel Bachem, Saskia Mitreuter, Yafit Levin, Jacob Y. Stein, Zhou Xiao, Zahava Solomon

**Affiliations:** ^1^ Bob Shapell School of Social Work and I‐Core Research Center for Mass Trauma Tel‐Aviv University Tel‐Aviv Israel; ^2^ Department of Medical Psychology and Medical Sociology University of Leipzig Leipzig Germany

## Abstract

Posttraumatic growth (PTG), the positive psychological transformations that follow traumatic events, affects both direct survivors (primary PTG) and their significant others (secondary PTG). Though primary and secondary PTG have been widely investigated in the literature, their long‐term trajectories decades after a traumatic event, especially as survivors enter older age, remain largely uninvestigated. Furthermore, it remains contested whether PTG adds up to a monolithic construct or rather consists of relatively independent components. Addressing these issues, we assessed a sample of Israeli male veterans from the 1973 Yom Kippur war (*N* = 349) and their wives (*N* = 156) at three time points over the course of nearly three decades. Both the veterans (primary survivors) and their wives (secondary survivors) reported PTG relating to the veterans' experiences during the war and/or captivity. Latent growth mixture modeling was conducted to identify trajectories of PTG on the five subscales of the Posttraumatic Growth Inventory. Long‐term trajectories of PTG followed heterogeneous patterns of fluctuation over time and particularly as participants entered older age. On most subscales, decreasing PTG scores were evident, a trend that was more pronounced among the primary survivors than the secondary survivors as primary and secondary PTG fluctuate considerably in the long‐term and seem to decrease as individuals enter older age. Furthermore, it would seem that PTG should not be considered a holistic concept but rather a conglomeration of positive changes. Implications of the findings are discussed within the context of limitations and potential intervening factors.

## Resumen

Spanish Abstracts by Asociación Chilena de Estrés Traumático (ACET)

Desarrollo longitudinal del crecimiento postraumático primario y secundario en veteranos ancianos y sus esposas: Trayectorias por dominios específicas

TRAYECTORIAS DE LOS DOMINIOS DE CRECIMIENTO POSTRAUMÁTICO

Tanto los sobrevivientes directos como sus parejas experimentan crecimiento postraumático (PTG, por sus siglas en inglés), las transformaciones positivas que siguen a los eventos traumáticos, vía PTG primaria (sobrevivientes) y PTG secundaria (parejas). Aunque el PTG primario y secundario han sido ampliamente investigados en la literatura, sus trayectorias de largo plazo décadas después de un evento traumático, y especialmente en la medida que los sobrevivientes adquieren edad avanzada, permanecen en gran parte sin investigar. Más aún, permanece controvertido si el PTG se agrega a un constructo monolítico o más bien está constituido por componentes relativamente independientes. Para abordar estos problemas, evaluamos una muestra de hombres veteranos israelíes de la guerra de Yom Kippur de 1973 (N = 349) y sus esposas (N = 156) en 3 puntos de tiempo en el curso de casi 3 décadas. Tanto los veteranos (sobrevivientes primarios) como sus esposas (sobrevivientes secundarios) reportaron PTG en relación a las experiencias de los veteranos durante la guerra y/o cautiverio. Se realizó un modelo de mezcla del crecimiento latente para identificar las trayectorias del PTG en las cinco subescalas del Inventario de Crecimiento Postraumático. Las trayectorias de largo plazo de PTG siguieron patrones heterogéneos de fluctuación a través del tiempo y particularmente en la medida que los participantes alcanzaban avanzada edad. En la mayoría de las subescalas, la disminución de los puntajes de PTG fue evidente, una tendencia que fue más pronunciada entre los sobrevivientes primarios que entre los sobrevivientes secundarios mientras que el PTG primario y secundario fluctua considerablemente en el largo plazo y parece disminuir mientras los individuos alcanzan avanzada edad. Más aún, pareciera que PTG no debería ser considerado un concepto holístico sino un conglomerado de cambios positivos. Las implicancias de los hallazgos se discuten dentro del contexto de las limitaciones y potenciales factores intervinientes.

## 抽象

Traditional and Simplified Chinese Abstracts by the Asian Society for Traumatic Stress Studies (AsianSTSS)

簡體及繁體中文撮要由亞洲創傷心理研究學會翻譯

Traditional Chinese

標題: 年紀漸長的退役軍人和他們的妻子原級和次級創傷後成長的縱貫發展: 範疇特殊的軌跡

撮要: 直接生還者與其身邊重要的人同樣會經歷創傷後成長(PTG)。那是創傷事件後的正向心理改變, 由原級PTG(生還者)和次級PTG(身邊重要的人)組成。雖然過往的文獻已廣泛地檢視原級和次級PTG, 可是, 兩者在創傷事件數十年後, 特別是當生還者踏入老年時的長遠軌跡, 仍缺乏研究。再者, PTG到底是否形成整體構念, 還是由相對獨立的成份組成, 仍備受爭議。為了解以上問題, 本研究檢視曾參與1973年贖罪日戰爭的以色列男性退役軍人(N = 349)及他們的妻子(N = 156), 在接近30年裡分3個時點進行評估。退役軍人 (原級生還者) 和妻子 (次級生還者) , 都有跟退役軍人戰爭和/或受虜經歷相關的PTG。潛在成長混合模型用以找出創傷後成長量表中5個子量表的PTG軌跡。PTG的長遠軌跡有不同的起伏模式, 特別是在樣本年紀漸長時。在大多數子量表裡, PTG分數都明顯下降, 而這趨勢在原級生還者當中更為明顯。長遠來說, 原級和次級PTG一直有顯著起伏, 並似乎因樣本年紀漸長而下降。而且, PTG似乎並非一個整體構念, 而是由一些正向改變結合而成。我們亦基於結果, 討論是次研究的限制和潛在的干擾因素。

标题: 年纪渐长的退役军人和他们的妻子原级和次级创伤后成长的纵贯发展: 范畴特殊的轨迹

撮要: 直接生还者与其身边重要的人同样会经历创伤后成长(PTG)。那是创伤事件后的正向心理改变, 由原级PTG(生还者)和次级PTG(身边重要的人)组成。虽然过往的文献已广泛地检视原级和次级PTG, 可是, 两者在创伤事件数十年后, 特别是当生还者踏入老年时的长远轨迹, 仍缺乏研究。再者, PTG到底是否形成整体构念, 还是由相对独立的成份组成, 仍备受争议。为了解以上问题, 本研究检视曾参与1973年赎罪日战争的以色列男性退役军人(N = 349)及他们的妻子(N = 156), 在接近30年里分3个时点进行评估。退役军人 (原级生还者) 和妻子 (次级生还者) , 都有跟退役军人战争和/或受虏经历相关的PTG。潜在成长混合模型用以找出创伤后成长量表中5个子量表的PTG轨迹。PTG的长远轨迹有不同的起伏模式, 特别是在样本年纪渐长时。在大多数子量表里, PTG分数都明显下降, 而这趋势在原级生还者当中更为明显。长远来说, 原级和次级PTG一直有显著起伏, 并似乎因样本年纪渐长而下降。而且, PTG似乎并非一个整体构念, 而是由一些正向改变结合而成。我们亦基于结果, 讨论是次研究的限制和潜在的干扰因素。

The concept of posttraumatic growth (PTG) denotes positive psychological transformations experienced by survivors of highly stressful and traumatic life events (Tedeschi & Calhoun, 2004). Posttraumatic growth has been documented in various populations in the immediate wake of traumatic events, such as violence (Kunst, 2010), sexual violence (Frazier, Conlon, & Glaser, 2001), and terror attacks (Levine, Laufer, Hamama‐Raz, Stein, & Solomon, 2008); it has also been found among former prisoners of war (POWs; Feder et al., 2008) and war veterans in general (e.g., Kaler, Erbes, Tedeschi, Arbisi, & Polusny, 2011).

Moreover, PTG has been documented in people who live in close contact with a trauma survivor. This phenomenon is termed “secondary PTG,” and it parallels the phenomenon of secondary traumatic stress (e.g., Figley, 1986). Secondary PTG has been documented among medical populations, such as parents of adolescent cancer survivors (Barakat, Alderfer, & Kazak, 2005) and husbands of breast cancer survivors (Weiss, 2004). In military populations, secondary PTG has been reported by wives of veterans (McCormack, Hagger, & Joseph, 2011) and wives of ex‐POWs (e.g., Greene, Lahav, Kanat‐Maymon, & Solomon, 2015).

Despite recent increased attention, fundamental questions regarding the nature of PTG remain unanswered, particularly: What trajectories does PTG follow decades after the trauma? And, what impact might aging have on long‐term PTG development? Additionally, it remains contested whether PTG is a monolithic construct or if it is better represented by its various subcomponents; this issue has yet to be investigated longitudinally. Moreover, these questions have rarely been considered with regards to secondary PTG. The aim of this study was to shed light on these issues by investigating changes in PTG and its substructures across nearly three decades in a sample of primary survivors, namely ex‐POWs and combat veterans along with a sample of secondary survivors consisting of the primary survivors’ wives.

Posttraumatic growth is one of several meaning‐making processes that begin in the wake of traumatic events (Park, 2010), and it is assumed to evolve over time. Its longitudinal trajectory, however, remains poorly understood. The majority of investigations of PTG have been conducted over a relatively short time period, spanning 2 years at most. Additionally, these studies have typically been conducted proximate to the traumatic episode and thus could not address the long‐term evolution. Theoretically, researchers have postulated that PTG increases over time as a result of cognitive and emotional processing of the trauma (Tedeschi & Calhoun, 2004; Tedeschi, Calhoun, & Cann, 2007; Zoellner & Maercker, 2006). The literature presents a more heterogeneous picture. Studies have documented increments in PTG across time (Frazier et al., 2009; McMillen, Smith, & Fisher, 1997) and group‐dependent increments (Thornton et al., 2012), but they have also shown decrements (Butler et al., 2005; Dekel, Solomon, Ein‐Dor, & Solomon, 2012). Nonetheless, in the majority of studies, researchers have found relatively stable rates of PTG throughout their measurements (e.g., Davis & Novoa, 2013; Frazier et al., 2001; Llewellyn et al., 2013; Silva, Crespo, & Canavarro, 2012).

To the best of our knowledge, only three studies have investigated the latent PTG trajectories with more than two assessment points. Marshall, Frazier, Frankfurt, and Kuijer (2015) investigated PTG trajectories at 1‐, 3‐, and 12‐month increments following two major earthquakes. Findings from the study revealed three stable profiles corresponding to high, moderate, and low growth. In contrast, two studies conducted among women suffering from breast cancer identified more complex trajectories. In one study, Wang, Chang, Chen, Chen, and Hsu (2014) identified four different trajectories of PTG over the course of one year: stable high, high decreasing, low increasing, and low decreasing. The second study, by Danhauer and colleagues (2015), was conducted over 2 years and revealed six trajectories: three stable, two modestly increasing, and one substantially increasing. Notably, no trajectories indicating PTG decrements were evident. Finally, Pat‐Horenczyk and colleagues (2016) investigated an integrated model of PTG at the start of treatment (baseline) and 6, 12, and 24 months posttreatment. Rather than highlighting PTG fluctuation, the researchers identified latent PTG classes based on measures of distress and coping. They traced trajectories of transition between the classes and found substantial fluctuation in PTG.

Notably, none of these studies distinguished trajectories of individual PTG components, and none focused on trajectories of secondary PTG. Therefore, although findings from the previously mentioned studies clearly indicated that PTG adheres to heterogeneous patterns of fluctuation, the manner in which primary and secondary PTG and their substructures wax and wane over longer periods of time remains unknown. As PTG and posttraumatic stress disorder (PTSD) are not mutually exclusive (Shakespeare‐Finch & Lurie‐Beck, 2014) and given the well‐established finding that PTSD fluctuates (e.g., Bonanno, 2004) even decades after the event (e.g., Solomon & Mikulincer, 2006), such an investigation is well warranted. This is particularly pertinent as trauma survivors enter older age.

The role of age in primary PTG has been the subject of two meta‐analyses that have focused on individuals suffering from life‐threatening illnesses. Findings have indicated that older age compared to younger age at the time of diagnosis is associated with less PTG (Helgeson, Reynolds, & Tomich, 2006; Shand, Cowlishaw, Brooker, Burney, & Ricciardelli, 2015). However, it remains unclear how PTG relates to the transition into older age among individuals who experienced a traumatic event at an earlier stage in life.

Considering the juxtaposing observations in the aging and trauma literature, two conflicting trends may be proposed. On one hand, aging has been associated with a preference for positive over negative information in attention and memory, termed the “positivity effect.” For instance, several studies have demonstrated that older adults reappraised negative events more positively than younger adults (Comblain, D'Argembeau, & Van der Linden, 2005; Rubin & Schulkind, 1997) and retrospectively evaluated their health more favorably than they had 14 years earlier (Kennedy, Mather, & Carstensen, 2004). Conversely, the socioemotional selectivity theory (Carstensen, Isaacowitz, & Charles, 1999) proposes that as people age and their time horizons shrink, a motivational shift takes place whereby older individuals increasingly focus on optimizing their emotional regulation and concentrate on positive aspects of an experience. Consequently, age‐related changes in time could be associated with changes in PTG in older adulthood. According to the socioemotional selectivity theory, as a person ages, the impact of trauma on his or her life story is remembered in a more positive light; therefore, an increase in PTG among traumatized aging individuals is to be expected.

On the other hand, the trauma literature proposes that older age is a high‐risk period for the development of PTSD (Davison et al., 2006) and thus may be associated with a decrease in PTG over time. For example, older age is often associated with a reduction in daily activities due to retirement and a reduced social network (e.g., Lee & Markides, 1990). Hence, more frequent opportunities emerge for reminiscing and life review (Erikson & Erikson, 1998). Research and clinical observations have indicated that with the increased time available to the trauma survivors to reminisce, and with fewer possibilities to distract themselves, traumatic memories often resurface and PTSD symptomatology is exacerbated or erupts in a late onset (e.g., Port, Engdahl, & Frazier, 2001). Increments in PTSD symptoms associated with aging, exacerbated by a trauma‐induced reduction in functioning and quality of life, may result in a reevaluation of growth perceptions previously held after the traumatic event. To the best of our knowledge, these two conflicting perspectives concerning PTG have not been explored in aging trauma survivors in respect to primary or secondary PTG.

Most PTG researchers have used the 21‐item Posttraumatic Growth Inventory (PTGI; Tedeschi & Calhoun, 1996), which assesses five components: improved relationships with others, realizing new possibilities in one's life, perceiving more personal strength, appreciating life more than before, and growing spiritually. Five PTGI subscales correspond with these factors and have been largely validated (e.g., Kaler et al., 2011). However, the utility of the measure as a whole, and specifically that of its constituting factors, is still contested (Christiansen, Iversen, Ambrosi, & Elklit, 2016). First, there are cultural variations in factor structure (Taku et al., 2007; Weiss & Berger, 2012). Second, many studies have only used the total PTGI score, despite the five subscales (e.g., Dekel et al., 2012; Silva et al., 2012), mostly because the subscales failed to contribute significantly to the overall understanding of the investigated phenomena. Finally, there is much debate about whether the construct is best understood as a unitary phenomenon made up of multiple and disparate factors or as being composed of several higher‐order factors (e.g., Joseph & Linley, 2006; Taku, Cann, Calhoun, & Tedeschi, 2008). These questions have been primarily addressed in the past with factorial analyses (e.g., Taku et al., 2008). However, exploring the long‐term trajectories of PTG may be of great value in this debate as it may determine whether these factors fluctuate in tandem or rather wax and wane in different directions.

By assessing veterans and their wives three times across 12 years, between 30 and 42 years after the war, our study aims were threefold: (a) to examine the long‐term trajectories of PTG in primary survivors (i.e., traumatized veterans) and secondary survivors (i.e., their wives), (b) to investigate whether the transition into older age is associated with increments or decrements of primary and secondary PTG, and (c) to explore the structure of primary and secondary PTG across time by evaluating latent trajectories of the PTGI's subscales.

## Method

### Participants and Procedure

For the present study, we utilized data from a longitudinal study focusing on the psychological implications of war among combat veterans and ex‐POWs from the 1973 Kippur War (for full details, see Solomon, Horesh, Ein‐Dor, & Ohry, 2012) and their spouses (for full details, see Greene, Lahav, Bronstein, & Solomon, 2014). To locate the veterans, we used Israel Defense Forces (IDF) records. Wives of veterans were recruited via their spouses. Both husbands and wives were contacted by telephone and asked separately to take part in the study. Questionnaires were administered in their homes or in another location of their choice. Before filling out the questionnaires, participants signed an informed consent form. This study was approved by the Tel Aviv University Institutional Review Board.

The veterans' data were collected at four time points: 1991, 2003, 2008, and 2015. The current study did not include data collected in 1991 as PTG was not assessed at that time. In the 1991 assessment, 520 veterans were contacted and 349 agreed to participate. Of these veterans, 287 participated in 2003 (Time 1 [T1]; 51 could not be located or refused, 5 had died, and 6 could no longer participate due to mental deterioration). In 2008 (Time 2 [T2]), the original 1991 veterans were recontacted and 289 participated (49 could not be located or refused, 25 had died, and 6 could no longer participate due to mental deterioration). In 2008, 82 veterans were added. In 2015 (Time 3 [T3]), 259 veterans took part (70 declined, 22 could not be located, 5 did not participate due to mental deterioration or other medical reasons, 2 did not return the questionnaire, 6 were abroad, and 48 had died). The veterans’ mean age was 53.5 years at T1 (*SD* = 4.6, range: 49–80 years), 56.8 years at T2 (*SD* = 5.0, range: 52–83 years), and 65.1 years at T3 (*SD* = 4.3, range: 59–80 years). The level of education in years did not change significantly from T1 (*M* = 13.9 years, *SD* = 3.4) to T3 (*M* = 14.0 years, *SD* = 3.9) and ranged between 8 and 25 years. At T3, 47.0% (*n* = 100) of participants were not working. The number of veterans who fulfilled PTSD criteria at T1, T2, and T3 was 82 (33.0%), 112 (50.0%), and 75 (35.3%), respectively. Veterans reported a mean of 8.3 (*SD* = 5.6, range: 0–23) physical health problems at T3.

Data from veterans’ wives were collected in 2003, 2010, and 2015. At T1, 213 of the 301 participating veterans were married or had a partner. Of this group, 156 (73.2%) wives participated. At T2, 250 veterans were married and 172 (68.8%) wives participated. At T3, 224 veterans were married and 133 (59.4%) wives participated. The wives’ mean age was 50.6 years at T1 (*SD* = 6.3, range: 42–72 years), 57.9 years at T2 (*SD* = 5.8, range: 46–79 years), and 64.6 years at T3 (*SD* = 5.3, range: 52–79 years). The wives’ level of education did not change significantly from T1 (*M* = 14.2 years, *SD* = 3.18) to T3 (*M* = 14.6 years, *SD* = 3.2, range: 8–24 years). At T3, 62.0 % (*n* = 83) were not working. At T1, T2, and T3 PTSD was endorsed among 22 (14.2%), 20 (15.6%), and 22 (15.3%) wives, respectively. Wives reported an average of 2.8 health problems at T3 (*SD* = 2.3, range 0–12).

### Measures

#### Life events

To assess negative childhood events, participants rated whether they experienced these events by 10 years of age: death of parent/sibling, parents' divorce, chronic disease with hospitalization, major economic difficulties, major accident, disability of a sibling, or any other relevant difficult situation. To assess participation in previous wars, veterans were asked whether they had participated in any Israeli wars prior to the Yom Kippur War. Life events after the war were measured using Solomon and Flum's (1988) Life Events Questionnaire, comprising 23 life events in four domains: family, work, health, and personal events. Participants rated whether they had experienced any event since the war until T3. We used the sum of negative life events after the war for analysis.

#### Battlefield stressors

A 21‐item self‐report questionnaire tapping the intensity of the fighting (e.g., “I saw a lot of dead soldiers,” “I killed enemy soldiers”) was used to assess battlefield stressors. Participants indicated the frequency of each on a 4‐point Likert scale ranging from 1 (*not at all*) to 4 (*I usually did*; Neria, 1993). Reliability value for this scale was high (Cronbach's α = .93).

#### Health problems

In 2008, both veterans and their wives completed a checklist of health problems (allergies, hypertension, ulcer, digestive problems, heart disease, chest pains, diabetes, malignant disease, weight gain, weight loss, back pain, headaches, joint pain, memory problems, and fatigue or weakness). The list covered common health problems affecting the central body systems as relevant for war veterans (Ohry et al., 1994). Respondents indicated on a dichotomous scale (*yes* or *no*) whether they suffered from the specific health problems. Adding up all positive answers, the total score represents the amount of health problems of a participant.

#### Posttraumatic stress disorder symptoms

The PTSD Inventory (Solomon et al., 1993) is a 17‐item self‐report scale assessing symptoms in the past month on a 4‐point scale ranging from 1 (*not at all*) to 4 (*I usually did*). A rating of 3 or above in any item was considered a symptom endorsement. Intensity of PTSD was calculated by the number of endorsed symptoms. Diagnosis was based on the *Diagnostic and Statistical Manual of Mental Disorders* (4th ed.; *DSM‐IV‐TR*; American Psychiatric Association, 1994), which was the standard when the study began. Reliability values for the scale's score were high at all assessments (Cronbach's αs = .91–.96 for total scores).

#### Posttraumatic growth

The PTGI (Tedeschi & Calhoun, 1996) was used to measure PTG, listing 21 items anchored with regard to the Yom Kippur War. Participants were asked to indicate on a 4‐point scale (1 = *I didn't experience this change at all* to 4 = *I experienced this change to a very great degree*) the extent of change that occurred in their lives following the war. The total score was computed according to the PTGI's five subscales: Relating to Others, New Possibilities, Personal Strength, Spiritual Change, and Appreciation of Life. The PTGI has demonstrated good reliability and validity (e.g., Kaler et al., 2011). Veterans’ Cronbach's alpha values were .96, .87, .88, .86, .79, and .75 at T1; .94, .79, .86, .88, .64, and .83 at T2; and .93, .90, .88, .78, and .82 at T3 for Improved Relationships, New Possibilities, Personal Strength, Spiritual Change, Appreciation of Life, and total score, respectively. In the same order of subscales, Cronbach's alpha values for the wives were .91, .85, .85, .88, .76, and .80 at T1; .89, .80, .86, .89, and .74 at T2; and .92, .88, .82, .86, and .83 at T3.

### Data Analysis

Couples were included if both veterans and their wives participated in at least one measurement (veterans, *n* = 246, 224, and 212, and wives, *n* = 154, 128, 133, at T1, T2, and T3, respectively). Overall, 8.9% to 56.1% of the data were missing. We used Little's Missing Completely at Random test to assess whether the missing values across waves were missing at random or if missingness was biased. Data were not missing completely at random, χ^2^(227, *N* = 246) = 1,441.480, *p* = .001. There were no demographic differences between individuals who did and did not participate. Missing data in all veterans' PTG factors were related to PTSD as those with high PTSD at T1 tended to participate in T2, but veterans with high PTSD at T2 tended not to participate in T3. Missing data were handled with robust maximum likelihood estimates for both spouses (Wang & Wang, 2012). Bivariate correlations were examined in SPSS (Version 24) between measures of life events and PTSD/PTG.

Trajectory analyses were conducted using Mplus 7.0 (Muthén & Muthén, 2012). We used unconditional latent growth mixture modeling, expecting heterogeneous subgroups with unique trajectories within a given population (Jung & Wickrama, 2008). Initially, we examined a series of unconditioned models (i.e., with no predetermined covariates) ranging from one‐ to four‐class solutions. As theoretical models of posttraumatic outcomes may entail curvilinear patterns, we included the intercept and the slope, both linear and quadratic. Fit indices were used to assess superiority of quadratic effects over linear ones. Variation in the slopes was tested to assess heterogeneous subgroups.

We used factor loadings that corresponded directly to the time intervals (i.e., setting the men's measurement points as 0, 5, and 12 and the women's as 0, 7, and 12). To avoid multicollinearity between the linear and quadratic slopes, we centered time points around the mean of the time scores (men, *M* = 5.66; women, *M* = 6.30) and set the linear time scores to 5.66, −0.66, and 6.34 in men and −6.33, 0.67, and 5.67 in women, respectively, at T1, T2, and T3. The quadratic time scores were 32.03, 0.43, and 40.19 in men and 40.07, 0.49, and 32.59 in women at T1, T2, and T3, respectively. Mean scores were divided by 10 to allow model convergence. The factor loadings of the intercept of the developmental trajectory of PTSD were fixed to 1.0.

To determine the optimal number of latent classes, solutions were evaluated based on fit statistics, interpretability, and theoretical considerations (Vermunt & Magidson, 2002). A good model fit is indicated by lower Bayesian information criterion (BIC), Akaike information criterion (AIC), higher entropy, significant Lo–Mendell–Rubin likelihood ratio test (LMR LRT), and significant bootstrap likelihood ratio test (BLRT). As many models were tested, we used Bonferronni correction for the BLRT and considered only *p* values lower than .001 to justify adding trajectories (Lo, Mendell, & Rubin, 2001; Nylund, Asparouhov, & Muthén, 2007). Fit indices were compared within linear models and between linear and quadratic models to assess superiority (lower AIC, BIC and adjusted BIC, improved entropy) over linear models (same number of classes) and examine whether LMRLRT and BLRT changed significantly. Next, we examined the extent to which PTSD may account for time‐specific change in PTG by using time‐varying covariates. When applying this method to latent growth mixture modeling (LGMM) to be a conditional LGMM with PTSD as time invariant covariate, the model did not converge so we estimated a conditional LGM with multiple growth process. We estimated a time‐varying covariate model in which indicators of PTSD at T1, T2, and T3 served as predictors of within‐time individual variability in five dimensions of PTG. This analytic strategy evaluates whether higher levels of PTSD at a particular time uniquely predict a time‐specific elevation in PTG dimensions beyond what is expected based on the individual‐specific trajectory of the measures. The goodness‐of‐fit values of this model were judged as satisfactory if the comparative fit index (CFI), and the Tucker‐Lewis index (TLI), were greater than .90, and root‐mean‐square error of approximation (RMSEA) was below 0.08 (Brown & Cudeck, 1992) with values less than 0.06 being preferred (Hu & Bentler, 1999).

## Results

### Associations Between Life Events, Battlefield Experience, and PTG/PTSD

Cross‐sectionally, no associations between life events and PTSD reached significance in veterans or wives, *r*s = −.07–.00, *p*s = .100–1.000. Correlations between PTG and life events were not significant, *r*s = .08–.03, *p*s = .281–.772. In veterans, higher battlefield stress reported in 1991 correlated with higher PTSD at T1, *r* = .27 *p* < .001; T2, *r* = .31, *p* < .001; and T3, *r* = .14, *p* = .011, as well as with higher PTG at T1, *r* = .26, *p* < .001; T2, *r* = .29, *p* < .001; and T3, *r* = .21, *p* = .003. As battlefield experiences were significantly associated with PTSD and PTG, we considered regressing PTG trajectories on battlefield experiences, but it would have reduced the sample size by half as data were anchored to participants with valid data in battlefield experience as assessed in 1991. Hence, we examined whether battlefield experiences were implicated in PTG trajectories, and all analyses of variance were nonsignificant (i.e., *p*s = .333–.746).

### Veterans' PTG Trajectories

Fit indices for linear‐only solutions for the veterans' PTG are presented in Table 1 with additional information for linear‐plus‐quadratic models of Spiritual Change and Personal Strength, the only dimensions in which these models converged. For Improved Relationships, BLRT was significant for the one‐class solution only, *p* < .001. Adding a second trajectory was not significant; thus, the third class was considered cautiously as it also yielded a small class without theoretical contribution. With the same criteria (BLRT < .001), the optimal classes for the remaining dimensions in linear models were two classes for New Possibilities, two classes for Spiritual Change, and three classes for Appreciation of Life. However, examination of linear‐plus‐quadratic models were superior (reduced AIC, BIC, and adjusted BIC in each number of classes). In the linear‐plus‐quadratic models, the Personal Strength two‐class option had significant LMRLRT, *p* = .034; and BLRT, *p* < .001, and the Spiritual Change three‐class option had a significant BLRT, *p* < .001. In the four‐class option of Spiritual Change, AIC, BIC, and adjusted BIC increased, so this option was excluded despite significant BLRT.

**Table 1:** Fit Indices for One‐ to Four‐Class Solutions for Veterans' Five Dimensions of Posttraumatic Growth (PTG)

Measure and Number of Classes	AIC	BIC	aBIC	Entropy	LMR LRT	BLRT
Improved Relationships						
**1**	**1,347.980**	**1,376.022**	**1,350.663**	–	–	–
2	1,346.821	1,385.380	1,350.510	0.588	6.750	6.750*
3	1,312.009	1,361.083	1,316.704	0.705	18.277	18.277***
4	1,318.009	1,377.599	1,323.710	0.766	18.277	18.277*
New Possibilities						
1	1,424.869	1,452.912	1,427.552	–	–	–
**2**	**1,403.757**	**1,442.316**	**1,407.446**	**0.663**	**27.112** **	**27.112** ***
3	1,400.929	1,450.003	1,405.624	0.706	8.829	8.829
4	1,406.929	1,466.519	1,412.630	0.767	0.000	0.000
Personal Strength (linear only)						
1	1,537.162	1,565.205	1,539.845	–	–	–
2	1,520.352	1,558.910	1,524.041	0.638	22.811*	22.811***
3	1,502.664	1,551.739	1,507.360	0.740	23.687*	23.687***
4	1,508.664	1,568.255	1,514.366	0.794	6.487	6.487
Personal Strength (linear and quadratic)						
1	1,492.89	1,515.20	1,501.31	0.66	41.15*	44.02**
**2**	**1,375.54**	**1,431.56**	**1,380.84**	**0.75**	**42.16** *	**44.08** ***
3	1,368.39	1,438.42	1,375.02	0.73	14.49	15.15*
4	1,375.54	1,431.56	1,380.84	0.75	13.64	14.25*
Spiritual Change (linear only)						
1	–	–	–	–	–	–
2	1,512.298	1,550.856	1,515.987	0.713	43.266*	43.266***
3	1,514.401	1,563.476	1,519.097	0.815	3.896	3.896
4	1,524.298	1,583.888	1,529.999	0.856	44.447	44.447
Spiritual Change (linear and quadratic)						
1	–	–	–	–	–	–
2	1,489.03	1,545.05	1,494.33	0.801	54.93	57.42
**3**	**1,426.79**	**1,496.82**	**1,433.42**	**0.860**	**67.18**	**70.23** ***
4	1,434.79	1,518.82	1,442.74	0.640	68.46	70.23***
Appreciation of Life						
1	1,604.691	1,632.734	1,607.374	–	–	–
2	1,579.052	1,617.611	1,582.741	0.712	31.639***	31.639***
**3**	**1,564.858**	**1,613.932**	**1,569.553**	**0.747**	**20.195** *	**20.195** ***
4	1,570.858	1,630.448	1,576.559	0.799	−2.379	−2.379

*Note*. Optimal solutions are marked in bold. AIC = Akaike information criterion; BIC = Bayesian information criterion; aBIC = adjusted BIC; LMR LRT = Lo–Mendell–Rubin likelihood ratio test; BLRT = bootstrap likelihood ratio test.

^*^ *p* < .05. ^**^ *p* < .01. ^***^ *p* < .001.

**Figure 1:**
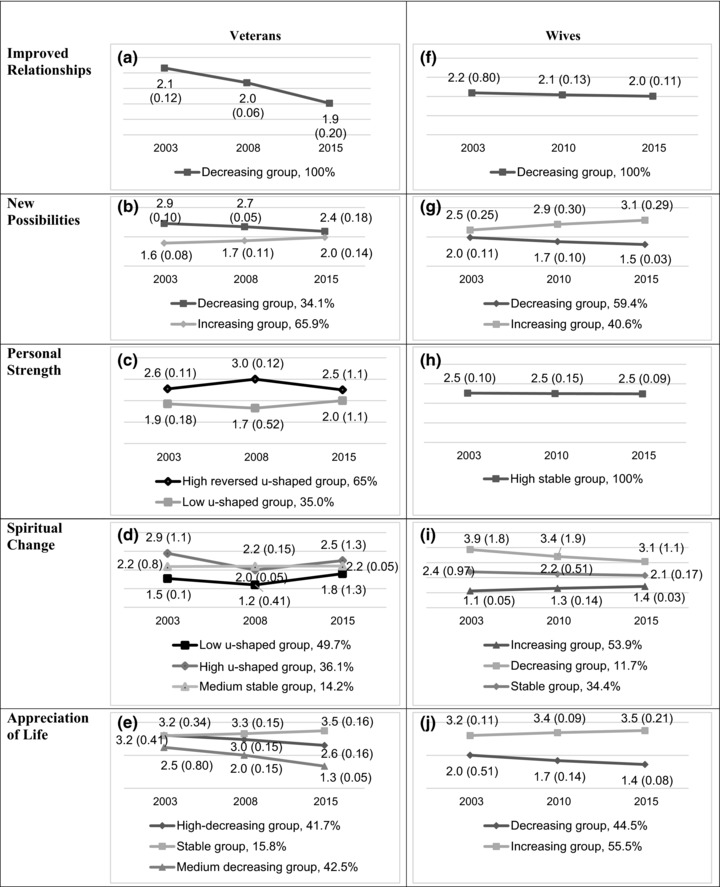
Posttraumatic growth (PTG) trajectories for veterans and their wives, according to PTG dimensions. Numbers in the graphs represent means and standard deviations.

Next, we examined the specific patterns of the various trajectories for the five PTG dimensions. Analysis of Improved Relationships (Figure 1a) showed a significant initial level and a significant negative unstandardized slope, intercept = 2.132, *p* < .001, *b* = −0.019, *p* < .001, and a nonsignificant correlation between initial improved relationship and change over time, *p* = .462. The trajectory was therefore defined as “decreasing Relationship Improvement group.”

For New Possibilities (Figure 1b), the unstandardized slopes for Classes 1 and 2 were negative and positive, *b* = −0.045, *p* < .01, intercept = 2.913, *p* < .001; and *b* = 0.033, *p* < .001, intercept = 1.567, respectively. In both classes, correlations between initial levels and slopes were significant, *r* = .20, *p* = .022, indicating that higher initial levels are associated with more change over time. Classes 1 and 2 were named, respectively, the “decreasing New Possibilities group” and the “increasing New Possibilities group.”

For Personal Strength (Figure 1c), the analysis revealed two classes that showed significant initial levels and significant linear and positive quadratic slopes, intercept = 1.85, *p* < .001, linear slope: *b* = −0.013, *p* < .001; and a significant positive slope, *b* = 0.01, *p* < .001. The second, smaller class had a significant intercept and significant linear and negative quadratic slopes, intercept = 2.61, *p* < .001, linear slope: *b* = 0.13, *p* = .008; and a negative quadratic slope, *b* = −0.01, *p* = .004. Accordingly, Class 1 was named “high reversed u‐shaped Personal Strength group” and Class 2 was named “low u‐shaped Personal Strength group.”

For Spiritual Change (Figure 1d), the analysis favored three classes. Class 1 had a significant intercept as well as both significant linear and positive quadratic slopes, intercept = 1.54, *p* < .001; linear slope: *b* = −0.17, *p* < .001; quadratic slope: *b* = 0.02, *p* < .001. Class 1 was named “low u‐shaped Spiritual Change group.” Class 2 had a significant high intercept as well as significant linear and negative u‐shaped quadratic slope, intercept = 2.89, *p* < .001, linear slope: *b* = 0.25, *p* = .001; quadratic slope: *b* = −0.03, *p* < .001. Hence, Class 2 was named “high u‐shaped Spiritual Change group.” Class 3 had a significant intercept but the unstandardized linear and quadratic slopes were not significant, intercept = 2.18, *p* < .001; linear slope: *b* = 0.00, *p* = .990; quadratic slope: *b* = 0.00, *p* = .600. Class 3 was named “medium stable Spiritual Change group." We found significant correlations between intercepts and linear slopes but not with quadratic slopes. The higher the intercept the higher the slopes, *r*s = −.15, −.20, and −.17, *p* < .001, for Classes 1, 2, and 3, respectively.

For Appreciation of Life (Figure 1e), we found negative slopes for Class 1, *b* = −0.051, *p* = .008, intercept = 3.215, *p* < .001; and Class 3, *b* = −0.095, *p* < .001, intercept = 2.482, *p* < .001, and a not significant unstandardized slope for Class 2, *b* = 0.025, *p* = .310, intercept = 3.188, *p* < .001. Class 1 was named “high‐decreasing Appreciation of Life group,” Class 2 was named “stable Appreciation of Life group,” and Class 3 was named “medium‐decreasing Appreciation of Life group.” The correlations between intercepts and slopes were nonsignificant, *p*s = .564–.993. All variations of slopes in all trajectories across all PTG subscales were significant.

### Veterans’ Wives' PTG Trajectories

Fit indices for one‐ to four‐class solutions for the wives' five PTG dimensions are presented in Table 2. For Improved Relationships, the analysis revealed that LMR and ALMR were significant only for the one‐class solution. Using the same criteria, the analysis revealed that the optimal classes for the remaining dimensions were two classes for New Possibilities, one class for Personal Strength, three classes for Spiritual Change, and two classes for Appreciation of Life. None of the models combining both linear and quadratic terms of the PTG factors converged.

**Table 2:** Fit Indices for One‐ to Four‐Class Solutions for Veterans’ Wives' Five Dimensions of Posttraumatic Growth

Measure and Number of Classes	AIC	BIC	aBIC	Entropy	LMR LRT	BLRT
Improved Relationships						
**1**	**794.192**	**818.488**	**793.167**	–	–	–
2	798.062	831.469	796.652	0.914	2.130	2.130
3	775.671	818.188	773.876	0.768	10.887	10.887
4	781.671	833.299	779.491	0.816	10.887	10.887
New Possibilities						
1	886.063	910.358	885.037	–	–	–
**2**	**866.210**	**899.617**	**864.800**	**0.683**	**25.852** *	**25.852** ***
3	859.325	901.842	857.530	0.659	12.885	12.885*
4	865.325	916.953	863.146	0.730	12.885	12.885
Personal Strength						
**1**	**939.538**	**963.833**	**938.512**	–	–	–
2	943.586	976.993	942.176	0.703	1.951	1.951
3	904.089	946.606	902.294	0.858	4.735	4.735
4	945.556	997.184	943.376	0.749	0.000	0.000
Spiritual Change						
1	858.538	882.834	857.512	–	–	–
2	830.102	863.509	828.692	0.843	34.436*	34.436***
**3**	**776.521**	**819.038**	**774.726**	**0.846**	**59.341** ***	**59.341** ***
4	788.616	840.244	786.437	0.907	53.486	53.486
Appreciation of Life						
1	962.859	987.155	961.834	–	–	–
**2**	**918.612**	**952.018**	**917.201**	**0.831**	**50.248** *	**50.248** ***
3	913.423	955.940	911.628	0.792	11.189	11.189*
4	930.612	982.240	928.432	0.916	−17.276	−17.276

*Note*. Optimal solutions are marked in bold. AIC = Akaike information criterion; BIC = Bayesian information criterion; aBIC = adjusted BIC; LMR LRT = Lo–Mendell–Rubin likelihood ratio test; BLRT = bootstrap likelihood ratio test.

^*^ *p* < .05. ^**^ *p* < .01. ^***^ *p* < .001.

Next, we examined the specific patterns of the trajectories for the five PTG dimensions. For Improved Relationships (Figure 1f), the analysis identified a significant unstandardized negative slope, *b* = −0.015, *p* = .005, intercept = 2.192, *p* < .001. The trajectory was therefore defined as “high‐decreasing relationship improvement group.” For New Possibilities (Figure 1g), the slope for Class 1 was negative, *b* = −0.040, *p* < .001, intercept = 1.961, *p* < .001; and the slope for Class 2 was positive, *b* = 0.056, *p* = .009, intercept = 2.470, *p* < .001. Class 1 was named “decreasing New Possibilities group” and Class 2 was named “increasing New Possibilities group.”

For Personal Strength, a significant initial level was revealed but no significant unstandardized slope, *b* = −0.003, *p* = .112, intercept = 2.523, *p* < .001. Accordingly, the trajectory was named “high‐stable Personal Strength group” (Figure 1h). For Spiritual Change (Figure 1i), a positive slope for Class 1, *b* = 0.025, *p* < .001, intercept = 1.113, *p* < .001; a negative slope for Class 2, *b* = −0.066, *p* = .001 and intercept = 3.871, *p* < .001; and a significant initial level and a nonsignificant slope for Class 3, *b* = −0.020, *p* = .211 and intercept = 2.386, *p* < .001, were revealed. The classes were respectively named “increasing Spiritual Change group,” “decreasing Spiritual Change group,” and “stable Spiritual Change group.” Considering Appreciation of Life (Figure 1j), a negative slope for Class 1, *b* = −0.047, *p* = .005 and intercept = 2.009 *p* < .001; and a positive slope for Class 2, *b* = .025, *p* = .310 and intercept = 3.203, *p* < .001, were revealed. Class 1 was named “decreasing Appreciation of Life group,” and Class 2 was named “increasing Appreciation of Life group.” All variations of the slopes in all trajectories across all PTG subscales were significant. No correlation between intercepts and slopes were significant, *p*s = .201–.999.

### The Role of PTSD in PTG Trajectories

The time‐varying covariates conditional LGM with multiple growth process showed acceptable model indices in veterans, χ^2^(204, *N* = 246) = 424.044, CFI = .931, TLI = 0.889, RMSEA = 0.066, 90% CI [0.057, 0.075]. The following estimates are standardized. In veterans, the results indicated that the higher the level of PTSD at T1, T2, or T3, the higher their Improved Relationships subscale scores at T1, β = .46, *p* < .001; T2, β = .41, *p* < .001; and T3, β = .18, *p* = .012, but this effect decreased with time. The higher the severity of PTSD at T1 and T2, the higher the New Possibilities score at T1, β = .36, *p* < .001; and T2, β = .19, *p* = .120. Level of PTSD at T3 had no significant effect on T3 New Possibilities scores, β = .15, *p* = .322. Severity of PTSD at T1 and T2 had significant effects on Personal Strength scores at T1, β = .30, *p* = .009; and T2, β = .31, *p* = .008, whereas T3 PTSD was unrelated to Personal Strength scores, β = .15, *p* = .334. Participants‘ ratings of Spiritual Change at T1 were related to PTSD only at T1, β = .23, *p* = .005, but not at T2 or T3, β = .14, *p* = .078 and β = .15, *p* = .280, respectively. Higher levels of PTSD at T1, T2, and T3 corresponded to higher ratings of Appreciation of Life, β = .31, *p* = .008 at T1; β = .45, *p* < .001 at T2; and β = .30, *p* < .001 at T3. Among the wives, PTSD severity measured at any time was unrelated to PTG scores or to changes in the dimensions of PTG.

## Discussion

Although health and clinical psychology have traditionally focused on the adverse effects psychosocial stressors may have on one's mental health, the emergence of positive psychology has included potential positive implications of traumatic events. The development and evaluation of concepts such as PTG have enriched the theoretical understanding of trauma and its aftermath as well as the spectrum of clinical interventions for trauma survivors. However, there is still much to be learned about the long‐term course of these concepts.

This study was the first to explore latent trajectories of individual PTG components longitudinally in adulthood. A sample of primary trauma survivors consisting of Israeli combat veterans and ex‐POWs along with a sample of secondary trauma survivors comprising the veterans' wives were assessed at three time points over 12 years. The results revealed diverse trajectories in most PTG domains, more often characterized by change, both increases and decreases, than stability over time. The finding that the various domains do not fluctuate in tandem but rather follow distinct trajectories indicates that the constituting factors of PTG correspond to distinct phenomena rather than a unitary construct. The results further suggest that as individuals age, PTG trajectories tend to decrease, tentatively suggesting that aging may be a risk factor for PTG development.

With regard to the long‐term trajectories of PTG, our results suggest that most aspects of PTG wax and wane over time for both primary and secondary survivors, albeit in varying directions. Among the veterans, a stable trajectory, exhibited only by 15.0% of the sample, was evident exclusively for the Appreciation of Life factor of PTG. In contrast, for the wives, a stable trajectory was the sole trajectory for Personal Strength and was evident also for approximately one‐third of the sample (34.4%) when considering Spiritual Change. These findings suggest that fluctuating trajectories of PTG are the norm rather than the exception for both primary and secondary survivors. This result is largely consistent with previous investigations of short‐term PTG trajectories among primary survivors (Danhauer et al., 2015; Pat‐Horenczyk et al., 2016; Wang et al., 2014), with the exception of Marshall et al. (2015), who identified only stable trajectories.

Overall, the primarily traumatized veterans reported declining PTG in more domains than their secondarily traumatized counterparts. On one hand, this could represent a more favorable longitudinal development of secondary PTG compared to primary PTG, which could be related to the fact that spouses’ trauma exposure was less severe. On the other hand, a potential variable of gender cannot be excluded as all primary survivors were male and all secondary survivors were female. As the fifth edition of the *DSM* (*DSM‐5*; American Psychiatric Association, 2013) expanded trauma exposure to include *s*econdarily traumatized significant others, it becomes valuable to explore whether their growth responses over time differ from primarily traumatized individuals and to further establish whether secondary traumatization can be equated to that of primary survivors (Horesh, 2016). Future studies should investigate PTG trajectories in both male and female individuals as well as primary and secondary survivors to disentangle the effects of exposure level and gender.

The finding that the various factors followed heterogeneous trajectories wherein both increases and decreases were evident provides a preliminary answer for the second objective of the study. It challenges the view that PTG is a unitary construct and suggests that it is rather an aggregate of qualitatively discernible positive changes. This observation is consistent with the finding that PTSD was not related with all PTG dimensions at all assessments. It is also consistent with findings from correlational studies that some variables, such as personality characteristics (e.g., Zoellner, Rabe, Karl, & Maercker, 2008), psychiatric symptoms (e.g., Zoellner & Maercker, 2006), or coping styles (e.g., Zhang, Yan, Du, & Liu, 2013) are associated with the different domains of PTG in unique ways. Although our findings do not necessarily impinge on the validity of the PTG concept or the PTGI as a measurement instrument, they strongly suggest that researchers and clinicians should focus not only on the total PTGI score but also consider its subscales separately.

From a theoretical perspective, aging could be related to both an increase in PTG in accordance with the socioemotional selectivity theory (Carstensen et al., 1999) or a decrease in PTG due to age‐related losses. The results show that among the veterans, PTG increase was the dominant pattern only in the domain of New Possibilities, whereas for the remaining subscales, decreasing PTG was more frequently indicated. Among the wives, increasing PTG scores were found to be the most frequent course for Spiritual Change and Appreciation of Life whereas Personal Strength remained stable and Improved Relationships and New Possibilities were characterized by decrease. Thus, in the current sample, a possible age‐related positivity bias did not distinctively manifest in increased PTG.

Conversely, results support the view that PTG components tend to decrease over time, which might be associated with age‐related losses regarding mental and physical health. Such losses include relinquishing one's previous daily routine and consequently having more time for reminiscing, which has been associated with delayed onset of PTSD symptoms (Buffum & Wolfe, 1995; Port et al., 2001). Indeed, previous studies with the present population identified a relatively high rate of delayed onset PTSD (Horesh, Solomon, Keinan, & Ein‐Dor, 2013). In addition to a decline in mental health, physical health and morbidity were also shown to be long‐term consequences of war trauma, as was shown in other populations (e.g., Beckham et al., 2003) as well as the present population (Solomon, Greene, Zerach, & Benyamini, 2014). The literature reports that several health indicators, declined immunity parameters, and lower perceived physical health are related to lower levels of PTG (see Barskova & Oesterreich, 2009, for a review). This association may be of particular importance for older adults as deteriorating health is a normative process that at some point becomes relevant to all older trauma survivors. It seems plausible that trauma‐related mental and physical deteriorations that typically only develop in older age, compounded by normative age‐related health declines, undermine perceptions of primary and secondary PTG over the lifespan.

However, the fact that there were increases and decreases evident in both primary and secondary PTG implies that PTG fluctuations cannot be explained with one unitary theory and do not conform to a single pattern as individuals enter older age. Rather, there are significant individual differences in the manners in which primary and secondary survivors' growth develops as time passes. This is especially noteworthy given that the measurements of PTG in the current study took place over nearly four decades after the initial traumatic experience and to date represent the longest longitudinal follow‐up of PTG trajectories.

The present study had several limitations. First, due to the attrition between measurements, the sample may be somewhat selective. Second, our initial measurement of PTG took place 30 years after the initial traumatic experience, and hence, we possess no information on PTG during the time before, immediately after, or in the first years following the trauma. Future studies should represent PTG development in a more continuous manner. Third, the current study used the PTGI, and it is possible that the use of other measures may yield different findings. Fourth, generalization of the results may be limited by the fact that Israel has remained politically tumultuous since its independence, continuing after the Yom Kippur War. Thus, participants remained exposed to recurring threats of varying degrees throughout the years of the study. It remains to be determined whether renewed exposure to traumatic events may impact longitudinal development of the components of PTG. Moreover, it is possible that results may differ after other traumatic experiences unrelated to war. Finally, various intrapersonal, interpersonal, and health‐related factors may be associated with PTG development; future studies should address the question of which factors predict the longitudinal course of different trajectories. In particular, PTSD is implicated in group‐level PTG, but in the current analysis, we could not determine its association with individual trajectories of PTG.

The limitations above notwithstanding, our findings have several clinical implications for the treatment of older trauma survivors. First and foremost, the current findings suggest that clinicians should not only assess for current PTG but also inquire as to its occurrence in the past. Furthermore, practitioners are advised to look at each PTG domain in its own right in this respect. Given the decrease in many domains of PTG among the aging participants, mental health professionals working with older trauma survivors are advised to pay particular attention to how their patients perceive different aspects of PTG and to potentially initiate interventions in order to foster favorable PTG development. Given that PTG was shown to be positively associated with a variety of indicators of successful aging such as cognitive functioning, interpersonal flourishing, and social support (e.g., Heckhausen, 2001; Ryff & Singer, 2000), this may be of particular relevance.
